# Egress Regulatory Factors: How *Toxoplasma* Exits from Infected Cells?

**DOI:** 10.3390/pathogens12050679

**Published:** 2023-05-04

**Authors:** Yujie Diao, Yong Yao, Saeed El-Ashram, Maohong Bian

**Affiliations:** 1 Department of Blood Transfusion, The First Affiliated Hospital of Anhui Medical University, Hefei 230032, China; 2 College of Life Sciences, Anhui Medical University, Hefei 230032, China; yaoyong@ahmu.edu.cn; 3 College of Life Science and Engineering, Foshan University, 18 Jiangwan Street, Foshan 528231, China; saeed_elashram@fosu.edu.cn; 4 Faculty of Science, Kafrelsheikh University, Kafr El-Sheikh 33516, Egypt

**Keywords:** *Toxoplasma gondii*, Egress, Pathogenesis

## Abstract

*Toxoplasma gondii* is an obligatory intracellular protozoan in the family Apicomplexa. It infects almost one-third of the world’s population and causes toxoplasmosis, a prevalent disease. The parasite’s egress from infected cells is a key step in the pathology caused by *T. gondii*. Moreover, *T. gondii*’s continuous infection relies heavily on its capacity to migrate from one cell to another. Many pathways are involved in *T. gondii* egress. Individual routes may be modified to respond to various environmental stimuli, and many paths can converge. Regardless of the stimuli, the relevance of Ca^2+^ as a second messenger in transducing these signals, and the convergence of various signaling pathways in the control of motility and, ultimately, egress, is well recognized. This review attempts to outline intra- and extra-parasitic regulators that mediate *T. gondii* egress, and provides insight into potential clinical interventions and research.

## 1. Introduction

*Toxoplasma gondii* is an intracellular parasite that can infect the nucleated cells (including immune cells) of almost all warm-blooded animals, including humans [1]. It is estimated that around 30% of the world’s population is chronically infected with *T. gondii* [2].

Although patients with chronic *T. gondii* infection may not exhibit clear clinical signs, multiple investigations have identified a possible link between *T. gondii* chronic infection and various mental diseases, including schizophrenia, bipolar disorder [3], and depression [4]. In chronic infection, *T. gondii* replicates slowly in the form of bradyzoites in tissue cysts in central nervous system (CNS) and muscles of people with competent immunity. However, in immunocompromised people (such as organ transplant recipients, AIDS patients, and others), pre-existing tissue cysts rupture, and the released bradyzoites may develop into tachyzoites, the cytolytic form of *T. gondii*, causing toxoplasmic encephalitis [5] and chorioretinitis [6]. Crucially, initial acute infection during pregnancy may lead to miscarriage, and congenital transmission has been linked to mental disorders, developmental problems, and deafness [7].

The life cycle of *T. gondii* alternates between asexual reproduction in intermediate hosts and sexual reproduction in definitive hosts. Tachyzoites, the major pathogenic stage of *T. gondii*, actively invade nucleated cells, multiply within host cells via endodyogeny, and egress from infected cells. *T. gondii*’s lytic cycle starts with active parasite invasion into host cells, which relies on protein release from two secretory organelles: micronemes and rhoptries [8]. In cats, the definitive hosts of *T. gondii*—unsporulated oocysts—are shed in faeces, and develop to the sporulated stage containing infective sporozoites. After consuming contaminated food or water, intermediate hosts become infected. After ingestion, oocysts develop into tachyzoites in the small intestine. Tachyzoites cause the acute stage of infection and evolve into tissue cyst bradyzoites. When the host’s immune system is suppressed, bradyzoites may either stay dormant for the duration of the host’s life or return to tachyzoites.

During *T. gondii* invasion, parasitophorous vacuole (PV) formation occurs through host cell membrane invagination (HCM) [9]. For *T. gondii* development, the PV membrane (PVM) acts as a molecular sieve, allowing tiny molecules to exchange passively. Proteins in dense granules (GRA) are discharged to construct the tubulovesicular network (TVN), which is a network of membranous tubules present throughout the PV, to maintain the nascent PV, which offers a safe niche for *T. gondii* proliferation [10]. *T. gondii* tachyzoites remain inside the PV until they egress, restarting the lytic cycle by attacking surrounding host cells. Egress was formerly assumed to be a simple passive process in which a nutrient-depleted host cell popped from inside, owing to the rising mechanical stress of the rapidly enlarging PV. The parasitic vacuolar membrane, the host plasma membrane, host endomembranous organelles (e.g., mitochondria, endoplasmic reticulum), and host cytoskeletal components must all be disrupted by the escaping parasites.

Host cell lytic cycle resulting from replication of tachyzoites leads to acute infection of *T. gondii*, which is characterized by damage to tissues and organs. Egress is seen as the initial phase. Exiting a parasite from a host cell is a damaging process that releases motile parasites. The gliding motility of extracellular tachyzoites is mediated through the interaction of microneme secretion and an actinomyosin motor. To initiate invasion, the parasite extrudes its conoid and adheres to the host cell at its apical end. This process is aided via microneme secretion. The parasite dwells in the parasitophorous vacuole formed via the invagination of the host membrane. During the invasion, the host mitochondria and ER are attracted to the parasitophorous vacuolar membrane. The parasites replicate until the egress signal causes egress, completing the lytic cycle. The genes that regulate egress are unclear, and the particular signals involved are unknown, although it is apparent that Ca^2+^ signaling is essential in mediating this process. Researchers realize that egress is a dynamic process in which the parasite employs various approaches, including intraparasitic egress signaling, and exogenous compounds [11,12]. These approaches will be addressed in the current review.

## 2. The Core Event of Egress: Ca^2+^ Signaling in *T. gondii*

To survive, *T. gondii* must adjust quickly and effectively to rapidly changing conditions, such as those encountered during invasion and egress. Ca^2+^ is a versatile second messenger that may work across a wide spatiotemporal range and control a wide range of essential cellular responses. *T. gondii* tachyzoites, akin to other eukaryotic cells, keep cytosolic Ca^2+^ at a significantly lower concentration (about 100 nM) than the external environment (in the mM range, a 10,000-fold difference) [13]. The coordinated activity of transport mechanisms in the plasma membrane and intracellular storage is closely controlled to maintain this sharp concentration gradient. Ca^2+^ release into the cytosol from intracellular storage or Ca^2+^ influx activates downstream effectors and propagates the signal along several pathways. As sustained high cytosolic Ca^2+^ concentrations are hazardous, a mixture of buffers, exchangers, and pumps immediately eliminated the calcium ion. Cells have, thus, developed regulatory mechanisms to control Ca^2+^ release and uptake in response to environmental stimuli. The location, amplitude, and frequency of these Ca^2+^ transients lead to many downstream effects. The endoplasmic reticulum (ER), acidocalcisomes, plant-like vacuole (PLV), and mitochondria are the vital intracellular depots involved in Ca^2+^ homeostasis in *T. gondii* [14,15]. The ER is thought to be the principal mobilizable source of Ca^2+^. Moreover, Ca^2+^ mobilization signals cause inositol 1,4,5-triphosphate (IP_3_) or ryanodine to bind to their respective ER receptors, causing Ca^2+^ to be released into the cytosol. Despite pharmacological evidence that IP_3_ and cyclic ADPR (cADPR) increase Ca^2+^ release from intracellular storage, there is no genetic support for the existence of an IP_3_ or ryanodine receptor homologue in *T. gondii* [16,17]. This complication shows that these receptors may differ significantly from those found in mammals. The extremely well-known and broadly conserved sarco-endoplasmic reticulum Ca^2+^ ATPase (SERCA) catalyzes Ca^2+^ absorption into the ER in *T. gondii* and is sensitive to thapsigargin, a tumor-promoting sesquiterpene lactone and inhibitor of SERCA-type Ca^2+^ ATPases [18,19]. Ca^2+^ leakage from the ER and inhibition of its re-uptake contribute to an increase in cytosolic Ca^2+^ resulting from thapsigargin inhibition [20,21,22]. Acidocalcisomes, characterized by their acidic nature, high density, and high amounts of pyrophosphate, polyphosphate, calcium, magnesium, and many other elements, also operate as necessary Ca^2+^ storage in *T. gondii* [23]. Acidocalcisomes include two proton pumps that participate in acidification: a vacuolar H^+^-ATPase (V-H^+^-ATPase) and a vacuolar H^+^-pyrophosphatase (V-H^+^-PPase), both of which have been reported and identified in *T. gondii* (TgVP1) [24]. A Ca^2+^ ATPase (TgA1) was also studied and found in both the acidocalcisome and plasma membrane of *T. gondii*. This protein helps move Ca^2+^ out of the cytosol [25,26]. There is also evidence that a Ca^2+^/H^+^ exchanger is involved in Ca^2+^ efflux. This suggestion is based on experiments that measured change in the proton gradient after Ca^2+^ was added. The acidocalcisome probably has a molar concentration of Ca^2+^; however, its main role seems to be storing Ca^2+^ since most of it is bound to polyphosphate and can only be released when the pH is more fundamental [14,27].

Ca^2+^ is also stored in *T. gondii* in apicoplast. This organelle possesses Ca^2+^/H^+^ exchanger functions, and the addition of glycyl-l-phenylalanine-naphthylamide (GPN) to extracellular parasites results in its breakdown with a cathepsin C protease, a rise in osmolarity, and a swelling effect that causes Ca^2+^ to leak into the cytoplasm [28]. There is additional evidence that TgA1 is present in the PLV, which has been linked to intracellular Ca^2+^ homeostasis [29]. Mammalian cells have a mitochondrial Ca^2+^ uniporter (MCU) that is activated via the electrochemical gradient produced via ATP hydrolysis, which promotes Ca^2+^ influx [30]. While *T. gondii* intracellular stores are shown to play a role in activating virulence features required for the lytic cycle, Ca^2+^ entry may also play a role in replenishing intracellular depots and promoting downstream effects triggered via high cytosolic Ca^2+^. Depletion of the ER activates store-operated Ca^2+^ channels in the plasma membrane, resulting in Ca^2+^ influx from the extracellular environment, known as store-operated Ca^2+^ entry (SOCE) [31]. However, there is no molecular evidence that Apicomplexan parasites have store-operated Ca^2+^channels (ORAI), ligand-operated channels, or second-messenger operated channels [15]. SOCE has been proven to be absent in *T. gondii* [32]. The existence of a nifedipine-sensitive Ca^2+^ entry route helps a voltage-gated Ca^2+^ channel function (VGCC). *T. gondii*’s genome contains many orthologues to VGCC and sequences that resemble transient receptor potential (TRP) channels [33,34]. Ca^2+^ influx and intracellular Ca^2+^ release both result in an increase in cytosolic Ca2+, activation of a variety of downstream processes, and the eventual return of cytosolic Ca^2+^ concentration to baseline levels, which is mediated through Ca^2+^-binding proteins. Ca^2+^-binding domains, such as EF-hand, allow signal transmission via these proteins. Calmodulin (CaM) from *T. gondii* was identified at both the apical end of external parasites and under the membrane of internal parasites, while the CaM inhibitors calmidazolium and trifluoperazine inhibited invasion [35,36]. Ca^2+^ signaling is widespread and vital in controlling many cellular activities, including *T. gondii* invasion and egress [37].

## 3. Intraparasitic Egress Signaling

### 3.1. Microneme Proteins

*T. gondii* active egress was originally linked to perforin-like protein 1 (TgPLP1), which plays a role in developing pores on PVM and HCM [38]. Gene knockout (KO) studies suggest that TgPLP1 pore formation disrupts PVM, which encases parasites during intracellular replication. PLP1-deficient parasites are delayed or fail in egress, and show a marked loss of virulence in infected mice, implying an association between efficient egress and virulence. Interestingly, both the N- and C-terminal domains of TgPLP1 have membrane-binding activity (dual-mode of membrane association), with the C-terminal domain (CTD) required for PVM permeabilization and lytic activity. The N-terminal domain of TgPLP1 also binds membranes and promotes rapid egress. Deleting the TgPLP1 N-terminal domain decreased parasite egress and lytic activity, but not virulence [39]. TgPLP1 CTD prefers binding lipids that are abundant in the inner leaflet of the lipid bilayer [40]. TgPLP1 CTD’s APCβ domain binds membrane phosphatidylethanolamine preferentially via a hydrophobic loop, which is aided via inositol phosphate lipids [41]. According to Marijo et al. [42], pH-dependent TgPLP1 membrane binding and cytolytic activity are pH-dependent, and pH-neutralizing drugs inhibit egress-associated membrane permeabilization. However, the precise intraparasitic control network involving acidification and TgPLP1 function is yet unknown. Exocytic vesicles mobilized via Ca^2+^ were recognized as lysosomes in mammalian fibroblasts and epithelial cells. It is widely known that *Trypanosoma cruzi*, the causative agent of Chagas disease, requires host lysosome recruitment for endocytic entrance into non-professional phagocytic cells [43]. Previous research showed that PVs containing *T. gondii* can host lysosome fusion and acidification [44,45], and some data show that *T. gondii* actively recruits host microtubules for the transport of host endo-lysosomes into the PV lumen in a time-dependent manner, with 73% of host cell lysosomes clustering around the PV two days after infection [46]. The distribution dynamics of parasite-derived proteins attached to PVM, and whether host lysosome sequestration aids parasite egress, remain unknown. Toxolysin 4 (TLN4), which is a structurally similar M16 family metalloproteinase, was the second microneme protein identified in *Toxoplasma* egress [47]. In mice, genetic disruption of TLN4 lowers the efficiency of egress from host cells, but does not affect virulence.

TgPLP1 and TLN4 are both in micronemes and have a calcium-dependent role in parasite egress [38,47]. However, these two effector proteins play different roles in parasitic virulence. The interaction between TgPLP1 and TLN4 necessitates further investigation. The identification of the functions of TgPLP1 and TLN4 was mainly based on the IIE system. The role of intra-parasitic calcium stores (such as the endoplasmic reticulum (ER) [48], acidocalcisomes [13], and the inner membrane complex [49]) and external calcium from the host cell should be determined. Though Carruthers et al. demonstrated that intracellular calcium reserves are sufficient to drive micronemal content discharge [50], more research into the particular calcium stores that trigger TgPLP1 and TLN4 release is required.

### 3.2. Dense Granule Proteins

The main functions of GRAs include changing the PV membrane (PVM) for nutrient acquisition from the host cell into the PV [51], the formation of TVN (as necessary for an antigen presentation) [34] and regulation of the host genome expression in the host cell’s nucleus [52]. Moreover, to facilitate parasite growth within PV, some GRAs or GRA-related proteins mediate the egress of *T. gondii*. Pingret et al. found that calcium is concentrated in the PV compared to the host cell cytoplasm, and the frequency of calcium sequestration rises before parasite egress, using non ratiometric markers in the PV to quantify calcium levels [53]. Interestingly, GRA41, which localizes within the PV and associates with the tubulovesicular network during *T. gondii* intracellular growth, affects the timing of parasite egress via regulation of calcium homeostasis in the PV [54]. Complete knockout of *GRA41* results in dysregulation of parasite calcium, which disrupts vacuolar morphology and alters the structure of the TVN. GRA41, unlike GRA1 [55], is a TVN component rather than a calcium-binding protein. GRA41′s function in calcium regulation might be explained by the fact that its deletion causes the PV lumen to shorten, resulting in a rise in calcium concentration in the PV. Sharp elevation of calcium concentration in PV triggers the discharge of microneme proteins, such as TLN4 and TgPLP1 [38,47], resulting in early egress of the GRA41 knockout strain [54]. A similar early egress phenomenon is observed when the GRA22 gene is genetically deleted in *T. gondii*; however, the absence of GRA22 does not influence the membrane region and intra-vacuolar network of PV [56]. GRA41 and GRA22 may employ distinct signaling pathways to control *T. gondii* egress. Following secretion, TgLCAT is a GRA-like protein that binds to the PV and parasite plasma membrane. *T. gondii* lacking TgLCAT shows delayed egress, whereas parasites over-expressing TgLCAT exit faster from infected cells [57]. Unlike conventional GRA proteins, which are secreted 10–30 min post-invasion and change the nascent PV [58], *T. gondii* synthesizes and releases TgLCAT from 7 h post-invasion until late in infection. In vitro, recombinant TgLCAT displays membrane lytic activity and dual enzymatic activity as a PLA2 and a cholesterol transacyltransferase, showing its potential to disrupt PVM. The upstream signal inducing TgLCAT expression is not yet clear; therefore, whether the accumulation of TgLCAT in PV lumen affects calcium flux through pore-forming on PVM is worth investigating.

Besides disrupting PV structure and altering calcium, some GRA functions as a component of the *T. gondii* cytoskeleton, and regulates parasite movement and egress. TgGRA8I is the first proline-rich protein found in the cytoskeleton of *Toxoplasma*. It links microtubules and actin filaments in the sub-pellicular region. Lack of the GRA8 gene alters the parasite morphology and the sub-pellicular cytoskeleton, and partially inhibits parasitic egress [59].

### 3.3. Calcium-Dependent Protein Kinases (CDPKs)

Calcium-dependent protein kinases (CDPKs), which are responders that use Ca^2+^ as a messenger, have been found in plants, ciliates and certain protists, but are absent from fungi and animals [60]. Due of their absence from their mammalian hosts, they are regarded as potential druggable targets. In *Plasmodium* spp., CDPKs play essential roles at various critical physiological stages during parasite development, including merozoite invasion and asexual intraerythrocytic development in humans and sexual/pre-erythrocytic development in *Anopheles* mosquitoes [61]. Currently, fourteen CDPKs have been identified that contribute to several functions in the biological processes of *T. gondii*, such as gliding motility, cell invasion, and egress [62]. TgCDPK1 is a crucial regulator of calcium-dependent microneme content release, including TgPLP1 [63]. TgCDPK1 knockout parasites did not rupture the PVM. Unlike TgCDPK1, which regulates exocytosis in *Toxoplasma*, TgCDPK3, which is found on the parasite’s periphery, controls *T. gondii* egress through particularly phosphorylated serines 21 and 743 of TgMyoA, which drives the parasite’s gliding motility [64]. Extracellular *Tgcdpk3*-*mutant* parasites display motility defects.

Collectively, we can provide a concise picture of how parasite proteins control *T. gondii* egress. After the invasion, *T. gondii* produces and secretes proteins critical for intracellular replication, including GRA41 for PV modification. *T. gondii* initiates TgLCAT production and releases TgLCAT into the PV lumen 7 h after the invasion. Accumulative TgLCAT uses its membrane lytic activity to create holes in the PVM, enabling calcium influx into the PV lumen from the host cell cytoplasm or calcium reserves in the host cell, such as mitochondria and ER. When calcium levels rise, micronemes release TgPLP1 and TLN4, which destroy PVM and HCM. However, damage to PVM and HCM by pore-forming proteins in microneme may not be sufficient for complete *T. gondii* egress. TgCDPKs phosphorate TgMyoA, which initiates parasite motility when calcium levels rise. *T. gondii* then completes its egress from infected cells. *T. gondii* egress occurs in three stages: (i) production and accumulation of TgLCAT; (ii) calcium influx from the host cell; and (iii) PVM and HCM damage, along with parasite motility.

Other parasitic own proteins that regulate egress of tachyzoites include preconoidal region protein 2 (Pcr 2) [65] and *Tg*EFP1 [66]. Pcr2 knockout parasites replicate normally, but they are severely diminished in their capacity for host tissue destruction due to significantly impaired invasion and egress. Knockout of *Tg*EFP1 results in faster propagation in tissue culture, hypersensitivity to calcium ionophore-induced egress, and premature natural egress (Table 1).

## 4. Extra-Parasitic Egress Signaling

### 4.1. Inflammatory Factors

*T. gondii* infection elicits host type I immune responses, characterized through the proliferation of specific CD8^+^ T cells and production of a variety of inflammatory cytokines, such as IL-12 and IFN-γ. These cytokines are vital for parasite control, intracellular replication, and activating host cells to remove parasites. Previous studies showed that externally triggered egress (ETE) is the main fate of *T. gondii* during acute infection in mice, which does not require reactive oxygen or nitrogen species, the mitochondrial permeability transition pore, or a variety of signal transduction mediators; instead, it depends on intracellular calcium and is highly sensitive to SB203580, which is an inhibitor of p38 MAPK and a related parasite-encoded kinase [67].

### 4.2. Gamma Interferon (IFN-γ)

IFN-γ induces PV disruption shortly after *T. gondii* invasion into murine astrocytes in an IGTP-dependent manner [68]. IFN-γ caused parasite egress and greatly decreased *T. gondii* replication in astrocytes in certain cases. In HFFs, IFN-γ induces cell death and early egress of *T. gondii*, which limits intracellular replication of *T. gondii* in HFFs and can promote clearance of the parasite by immune cells. IFN-γ-induced egress does not depend on the deprivation of tryptophan or iron [69]. However, another study using IFN-γ-stimulated MEFs as host cells found that the rupture of the PV resulted in the death of *T. gondii* instead of egress [70]. Cell lines used as host cells in all three models were stimulated after IFN-γ pretreatment for varying durations. Thus, *T. gondii* egress was a direct consequence of PV disruption resulting from effectors involved in IFN-γ signaling, although the role of parasitic motility and intraparasitic calcium needs future investigation. *T. gondii* can infect and multiply intracellularly in “naive” cells that inflammatory cytokines have not triggered. It is currently unknown if IFN-γ post-treatment causes *T. gondii* egress.

### 4.3. Nitric Oxide (NO)

One variable related to tachyzoite and bradyzoite interconversion was NO (a mitochondrial function inhibitor) [71]. NO released by activated microglia is toxic to neurons and leads to apoptosis of neuron cells [72]. Two in vitro studies showed exogenous NO released by sodium nitroferricyanide (III) dihydrate (SNP) could trigger the egress of *T. gondii* tachyzoites from infected peritoneal macrophages and HFFs [73,74]. The occurrence of NO-induced egress depended on intraparasitic calcium levels and the mobility of *T. gondii* [73]. However, no evidence suggests that NO induces egress of *T. gondii* from host cells in vivo.

### 4.4. Death Receptor and Perforin

Death receptor ligation and the perforin-dependent granule exocytosis are two pathways used by cytotoxic cells to induce apoptosis of target cells, such as pathogen-infected cells. A study showed that death receptor ligation in *T. gondii*-infected cells causes rapid egress of tachyzoites and lytic necrosis of the host cell, which is mediated via the release of intracellular calcium because of caspase activation early in the apoptotic cascade. More importantly, upon acting on infected cells via death receptor- or perforin-dependent pathways, T cells induce rapid egress of *T. gondii* capable of infecting neighboring cells, including the Ag-specific effector cells [75]. Death receptor- and perforin-mediated parasite egress may contribute to parasite dispersion in peripheral tissues and systemically during *T. gondii* infection. Active macrophages or other immune cells may engulf and kill free tachyzoites because of this death receptor- and perforin-mediated parasite egress.

### 4.5. Tumor Necrosis Factor-α (TNF-α)

TNF-α is a key inflammatory factor in controlling *T. gondii* dissemination [76]. An in vitro study reported that high doses of TNF-α– induced egress of *T. gondii* from infected HFF cells in a time-dependent manner. Blocking the host apoptosis pathway significantly decreased TNF-α– induced egress [77]. However, this finding cannot mimic the in vivo situation because the concentration of TNF-α used in this study was higher than that of in vivo conditions. 

## 5. Exogenous Compounds

Besides inflammatory factors, various exogenous compounds were used to investigate the processes of *T. gondii* egress. According to previous research using ionophores to artificially stimulate egress, increasing cytoplasmic Ca^2+^ stimulates microneme production, motility, and egress. Using a Ca^2+^ ionophore—A23187—and an ionophore-resistant mutant, the link between Ca^2+^ and egress was first proven [78]. Ionophore-induced egress is thought to follow the same signaling pathways as natural egress since it matches the fast egress of intracellular parasites. Before egressing, the tachyzoites showed morphological alterations and movement, suggesting that the cytoskeleton and motility machinery are required for this process. Ionophore-resistant mutants also displayed a deficiency in host cell permeabilization before parasite egress, showing that cell permeabilization was a critical step in the parasite egress signaling pathway [79]. The mutants that had a delay in ionophore-induced egress also had a delay in ethanol-induced egress [80]. Ethanol was initially thought to trigger phospholipase C (PLC) activity, resulting in a rise in inositol 1,4-5-triphosphate (IP3), the release of Ca^2+^ from intracellular reserves, and the stimulation of extracellular parasite microneme secretion [81]. The application of an IP3 inhibitor also prevented microneme secretion, adhesion, and invasion [17]. These findings show that ethanol similarly causes egress via Ca^2+^ ionophores through increasing Ca^2+^ fluxes, and that the same intracellular reserves engaged in microneme production and invasion may also be involved in egress.

Other investigations have shown that permeabilization of the host cell happens immediately before natural egress. When membrane integrity is lost, ions and small molecules exchange between the intracellular and external environments, exposing parasites to these alterations. The significance of K^+^ in egress was highlighted using an artificial permeabilization system that exposed intracellular tachyzoites to buffers of various ionic compositions [82]. On host permeabilization, the low concentration of K^+^ in the extracellular environment was postulated to activate a parasite PLC, which would then increase the parasite’s cytoplasmic Ca^2+^ and, eventually, events prompting egress. The ionophore nigericin, which selectively causes efflux of potassium from the cell, effectively causes egress of *T. gondii* within 30 min of treatment with that drug, which requires phospholipase C function and parasite motility [83]. This work showed that nigericin-induced egress relies on Ca^2+^ signaling and motility, implying that host cell K^+^ loss occurs upstream of intraparasitic Ca^2+^ fluxes on the egress route. The intracellular environment’s high K^+^ content has been linked to regulating microneme production and motility, both controlled via Ca^2+^ release from intracellular reserves and essential for parasite egress from the host cell. Acidification of the parasitophorous vacuole has been shown to overcome the inhibition of high K^+^ levels in the intracellular environment, enhancing motility and microneme secretion [42]. Perforin-like protein 1 (PLP1), which is a pore-forming protein that inserts itself into membranes at low pH and is crucial for lysis of both the PVM and the host plasma membrane, permitting egress, is a microneme protein of special interest. Upon ionophore stimulation, PLP1-deficient parasites exhibited an egress defect, trapping inside the host cells and reducing pathogenicity [38,39,50]. This result supports the concept that an egress is a parasite-controlled event, rather than a passive one resulting from host cell rupture owing to reaching maximum capacity.

Using dithiothreitol (DTT), which is a reducing chemical that stimulates parasite nucleotide triphosphate-degrading enzymes (NTPs) released into the PV, is also shown to promote *T. gondii* egress [84,85]. Dithiothreitol (DTT) added to the culture medium outside *T. gondii*-infected host cells stimulated motility and egress of intravacuolar parasites, invariably accompanied by a detectable Ca^2+^ flux within the host cell [84]. When the enzyme is reduced, there is a decrease in ATP in the host, and a rise in cytoplasmic Ca^2+^ just before egress. Uninfected host cells and extracellular parasites do not display an increase in Ca^2+^, and only infected host cells show a drop in ATP when stimulated with DTT, reinforcing the response’s reliance on both the host and parasite, and suggesting that Ca^2+^ flow and NTPase activity may be connected. The NTPases are thought to cause the disrupted Ca^2+^ ATPases in the host ER, resulting in an increase in Ca^2+^ in the cytosol around the parasite. It is still unclear whether the mechanism causes NTPase activation in vivo, or if their lowering serves as a critical egress sensor.

The accumulation of the phytohormone abscisic acid (ABA), continually generated by the parasites during intracellular reproduction, was also examined as an egress route [86]. The parasite is thought to egress whenever the concentration of ABA exceeds a particular threshold. In response to environmental stress, ABA causes the dose-dependent synthesis of the second messenger cyclic ADP ribose (cADPR), which releases intracellular Ca^2+^ in *T. gondii* and plants [87]. The exact mechanism via which ABA is detected remains unknown; however, the data show that the lack of ABA leads to quiescent intracellular parasites, while chelation of intracellular calcium blocks ABA-induced microneme production.

## 6. Future Perspective

Egress is a critical stage in the intracellular lytic cycle of *T. gondii*. This mechanism causes inflammation and is intimately linked to the pathogenesis of toxoplasmosis. There are flaws in the existing in vitro models for egress. The HFF line is most commonly employed in both traditional cultures and in vitro investigations. This method is used mostly for convenience since these cell lines are simple to maintain in culture, and their flat shape makes them perfect for microscopy. However, this cell type is unlikely to be infected with *Toxoplasma* during a natural infection. One alternative would be to perform egress assays in a context that more accurately reflects what we observe in vivo studies, namely through selecting immune cell lines or nerve cells to replace HFFs, and performing these assays in inflammatory conditions that mimic the severe inflammatory immune response that is characteristic of *T. gondii* infection. Current approaches for examining egress depend heavily on activating intracellular calcium signaling in the parasite to induce egress of intracellular parasites. Interactions between CD8 T cells and infected cells, for example, may be a trigger for parasite egress. This approach is probably a means for the parasite to detect and react correctly when its environment is under attack. At least two mechanisms describe this event: signaling through Fas ligand and perforin attack. An effective way of examining egress in vitro would be via a biological method, rather than pharmacological stimulation of egress. Infected cells that have been altered to express the correct receptors, or selected for endogenous expression of these molecules, might be stimulated via the addition of recombinant ligands or activated immune cells that express these molecules. This induction tactic would be a better approach to studying egress because it would more accurately mimic the actual events that stimulate egress during toxoplasmosis. It would also induce a response that uses relevant signaling pathways and, presumably, elicits responses at a natural level. Egress has always been challenging to examine due to how little is understood about the process and its dynamic nature. A more refined approach might enable future research to find phenotypes related to genes with minimal influence. In the future, organoid tissues may be used in the egress study of *T. gondii* in more complex systems.

## Figures and Tables

**Table 1:** *T. gondii* effector proteins regulating parasitic egress from host cells.

Protein	Cellular Location	Function	Ref.
TgPLP1	Microneme	Permeabilize the PVM and HCM	[38]
TLN4		Putative metalloproteinase	[47]
GRA8	Dense granule/PV lumen	Component of the sub-pellicular cytoskeleton	[59]
GRA22		N/A	[56]
GRA41		Regulation of calcium homeostasis	[54]
TgLCAT		Potential to disrupt membranes	[57]
TgCDPK3	Parasite periphery	Phosphorylation of TgMyoA	[64]
TgCDPK1	N/A	Regulation of microneme secretion	[63]
Pcr 2	Preconoidal region	----	[65]
TgEFP1	PLV/ELC and the PV	----	[66]
